# β-Cell Regeneration Mediated by Human Bone Marrow Mesenchymal Stem Cells

**DOI:** 10.1371/journal.pone.0042177

**Published:** 2012-08-07

**Authors:** Anna Milanesi, Jang-Won Lee, Zhenhua Li, Stefano Da Sacco, Valentina Villani, Vanessa Cervantes, Laura Perin, John S. Yu

**Affiliations:** 1 Division of Endocrinology, Cedars-Sinai Medical Center, Los Angeles, California, United States of America; 2 VA Greater Los Angeles Healthcare System, Los Angeles, California, United States of America; 3 Department of Neurosurgery, Cedars-Sinai Medical Center, Los Angeles, California, United States of America; 4 Department of Urology, Children’s Hospital Los Angeles, University of Southern California, Los Angeles, California, United States of America; Mayo Clinic, United States of America

## Abstract

Bone marrow mesenchymal stem cells (BMSCs) have been shown to ameliorate diabetes in animal models. The mechanism, however, remains largely unknown. An unanswered question is whether BMSCs are able to differentiate into β-cells *in vivo*, or whether BMSCs are able to mediate recovery and/or regeneration of endogenous β-cells. Here we examined these questions by testing the ability of hBMSCs genetically modified to transiently express vascular endothelial growth factor (VEGF) or pancreatic-duodenal homeobox 1 (PDX1) to reverse diabetes and whether these cells were differentiated into β-cells or mediated recovery through alternative mechanisms. Human BMSCs expressing VEGF and PDX1 reversed hyperglycemia in more than half of the diabetic mice and induced overall improved survival and weight maintenance in all mice. Recovery was sustained only in the mice treated with hBMSCs-VEGF. However, de novo β-cell differentiation from human cells was observed in mice in both cases, treated with either hBMSCs-VEGF or hBMSCs- PDX1, confirmed by detectable level of serum human insulin. Sustained reversion of diabetes mediated by hBMSCs-VEGF was secondary to endogenous β-cell regeneration and correlated with activation of the insulin/IGF receptor signaling pathway involved in maintaining β-cell mass and function. Our study demonstrated the possible benefit of hBMSCs for the treatment of insulin-dependent diabetes and gives new insight into the mechanism of β-cell recovery after injury mediated by hBMSC therapy.

## Introduction

Despite studies showing that strict blood glucose control decreases the incidence of secondary complications of diabetes, euglycemia is difficult to achieve with current methods of exogenous insulin replacement. Although transplantation of the whole pancreas or islets of Langerhans demonstrates the physiologic advantages of transplanting insulin-producing cells over insulin administration, these approaches are far from perfect. Ideally there are two possible solutions: identifying the perfect “surrogate β-cells” to be used for cell therapy or inducing regeneration of endogenous damaged β-cells.

BMSCs offer an attractive source of stem cells as an alternative to pancreas and pancreatic islet transplantation for curative and definitive treatment of insulin-dependent diabetes. Proven to be safe in clinical trials, BMSC can be obtained with relative ease from each patient, allowing potential circumvention of allograft rejection. Previous work suggested that mouse BMSCs spontaneously differentiate into endocrine pancreas cells in vivo [1]. In recent reports, BMSCs injected into the circulation of diabetic animals have been shown to partially/totally reverse the diabetic phenotypes and improve glucose control [2], [3], [4], [5], [6], [7], [8] [9] [10], [11] but with very poor direct β-cell differentiation, leading to other possible roles of BMSCs in pancreatic islet regeneration. Additionally, we previously reported that manipulation of culture conditions could induce β-cell differentiation in rat BMSCs expressing nestin [12]. We were able to induce differentiation of bone marrow stem cells into all three neural phenotypes [13], [14], and pancreatic endocrine cell lineage [12], suggesting the existence of a common precursor for both neuronal and islet cell types in the bone marrow.

Introduction of transcription factor genes into cultured human BMSCs was able to activate a number of genes related to the development and function of β-cells [15], [16]. Moreover, the forced expression of PDX1 gene by a viral vector in human BMSCs clearly showed the activation of gene expression of all four islet hormones and also enhancement of significant insulin content [17]. These data propose the possibility of a new paradigm for treating diabetes using a patient’s own stem cells. Multiple questions still need to be answered: Can BMSCs differentiate into functional β-cells? And, can BMSCs mediate the recovery of injured endogenous β-cells *in vivo*, and how? In order to approach these questions, we used mesenchymal stem cells isolated from human bone marrow (hBMSCs) since they have been reported to induce partial reversion of hyperglycemia in a diabetic animal model [7]. In order to identify a possible mechanism for β-cell differentiation or β-cell recovery in the context of stem cell therapy, we compared the effect of two genes, PDX1 and VEGF on hBMSCs. PDX1 is a well known master gene in pancreas development and crucial for early pancreatic differentiation [18] and later in β-cell fate determination [19]. VEGF-A is an important factor for intra-islet angiogenesis [20], and the vascular membrane is a niche for insulin gene expression and β-cell proliferation [21]. It has been suggested that relative loss of intra-islet vascularization after streptozotocin administration limits subsequent β-cell regeneration [22]. Interestingly, islet cells expressed several angiogenic factors including VEGF from early development throughout adulthood in mice [23]. Despite having normal pancreatic insulin content and β-cell mass, β-cells with reduced VEGF-A expression showed impaired insulin secretion after glucose stimulation in mice [20]. Moreover, VEGF increased pancreatic islet survival after islet transplantation by stimulating intra-islet revascularization [24], [25].

Therefore, we used hBMSCs, hBMSCs expressing PDX1, and hBMSCs expressing VEGF as treatment groups. Our results showed that human BMSCs have a pronounced plasticity *in vivo* and can be differentiated into multiple cell types including insulin producing cells with the aid of transient expression of external genes. Surprisingly, detectable levels of human insulin were present in mice in both cases, treated with either hBMSCs-VEGF or hBMSCs-PDX1. However, clinically sustained reversion of diabetes was obtained only after transplantation of hBMSCs-VEGF suggesting that the recovery was secondary to endogenous β-cell regeneration. VEGF could play a key role in supporting engraftment of hBMSCs and their differentiation into vasculature in the diabetic pancreas *in vivo*. Bone marrow stem cells can possibly act as a bridge, enabling delivery of nutrients and oxygen to damaged endogenous β-cells. Therefore, our system provides new insight into the mechanism of pancreatic islet neogenesis, which can be translated into therapy of insulin-dependent diabetes.

## Research Design and Methods

### Human BMSC Culture and Expansion

BMSCs from a normal adult donor were purchased from a commercial source AllCells, LLC (5858 Horton Street, Suite 360, Emeryville, California, 94608 USA; catalog number ABM005). All of nucleated cells were plated into a basal medium consisting of Alfa-MEM, 17% fetal bovine serum, 2 mM glutamine, 50 U/L penicillin and 25 µg/L streptomycin (all from Invitrogen). After 24 hr in culture, non-adherent cells were separated from adherent ones. The adherent cells were washed with PBS and cultured with the previous medium for 5–7 days. At 80% confluence adherent cells were harvested with trypsin/EDTA and plated in new dishes (passage 1). Cells underwent two further expansions before aliquots were suspended in 90% FBS and 10% DMSO, and frozen at −80°C. Until passage number (#) 6–7 the cells grew with a doubling time of 48 hrs. All experiments were performed using a single batch of hBMSCs from a single donor. The cells were used within passage #7. Human BMSCs from passage # 3 to 6 were analyzed for various cell surface markers commonly used for the positive and negative detection of mesenchymal stem cells by flow cytometry analysis (CD44, CD31, CD34, and CD 105). To characterize the mesenchymal potential of hBMSCs, we induced adipogenic and osteogenic differentiations *in vitro* (data not shown).

### Adenovirus Production and Cell Transfection

cDNAs encoding for human Pdx1 and mouse VEGF165 (kindly donated by Dr. Patricia A. D'Amore, Schepens Eye Research Institute, Harvard medical school to Dr. Laura Perin, Children Hospital Los Angeles) were subcloned into Adeno-X viral DNA vector (BD Biosciences Clontech), following manufacture protocol. CMV was used as promoter. Successful homologous recombination resulted in recombinant virus encoding for PDX1 (Ad-PDX1) and VEGF (Ad-VEGF). The viruses were expanded in HEK293 cells as described in the ViraPower Adenoviral Expression system manual from Invitrogen. Human BMSCs were transfected with adenovirus carrying PDX1 (hBMSC-PDX1) or VEGF (hBMSC-VEGF) 2 days before transplantation. RNA and protein levels of PDX1 and VEGF in transfected cells were assessed by PCR and Western Blotting.

### Animal Model and Stem Cell Transplantation

To induce diabetes, NOD/SCID mice (The Jackson Laboratory) 6–8 weeks of age were given three intraperitoneal injections of streptozotocin (STZ) [Sigma-Aldrich, Saint Louis, MO], 50 mg/kg, on day 1–3. All experiments and procedures were performed according to an approved protocol by the Institutional Animal Care and Use Committee at Cedars-Sinai Medical Center. One healthy control group did not receive any treatment. STZ treated groups were divided into 4 groups: one received a sham injection after induction of diabetes with STZ, and the other 3 groups received hBMSCs, hBMSCs-PDX1, or hBMSCs-VEGF. Additionally, two groups of STZ treated mice were transplanted with mouse fibroblasts transfected with adenovirus expressing PDX1 or VEGF.

On day 0, about 7 days from STZ treatment, mice showing hyperglycemia (glucose level >250 mg/dl) were transplanted with about 1×10^6^ cells each. To avoid aggregation of the cells, cells were thoroughly suspended in 150 µl and injected with a 30 gauge needle through the chest wall into the left cardiac ventricle as previously described [7]. The animal weights were recorded on the day of bone marrow transplantation and on the last day of the study. All animals were sacrificed to harvest peripheral blood and tissues at 6 weeks after cell transplantation. Achievement of normoglycemia was defined as blood glucose <200 mg/dl.

### Blood Glucose and Serum Insulin Measurements

Blood glucose was measured in non-fasting mice between 9 and 11 am daily for the first week than two times a week. The level of glucose was measured from the tail vein using One Touch Ultra Meter and Test Strips (Lifescan Inc., Milpitas, CA). The sensitivity of the assay does not exceed 600 mg/dl, and thus the maximal extent of hyperglycemia can be over the limit. Mouse and human serum insulin levels were determined by ultrasensitive mouse insulin enzyme-linked immunosorbent assay (ELISA) (Alpco Diagnostics, Salem, NH) and human insulin ELISA (Linco Research, Millipore Corporation, Billerica, MA), respectively according to the manufacture protocols at 6 weeks after stem cell injection. Three replicates were performed for each sample.

#### Immunohistochemical analyses

The mouse pancreatic tissues were harvested 6 weeks after stem cell injection and immediately fixed with 4% paraformaldehyde at 4°C overnight. The tissues were then dehydrated in graded ethanol, cleared in xylene and finally embedded in paraffin. For immunohistochemical staining of the paraffin embedded samples, sections were deparaffinized in xylene and rehydrated through ethanol baths and PBS, followed by rinsing in distilled water for 5 min. Pancreatic sections were stained in Harris hematoxylin solution and eosin Y solution (Sigma). For immunofluorescent staining, antigen retrieval was performed by heating at 90°C in antigen retrieval buffer (DAKO). Pancreatic islets were stained with various primary antibodies: mouse monoclonal anti glucagon (Sigma-Aldrich, dilution 1∶100), mouse monoclonal anti VEGF (Novus Biological, dilution 1∶100), rabbit polyclonal anti insulin (Santa Cruz, dilution 1∶50), rabbit polyclonal anti-p27Kip1 (Abcam, dilution 1∶200), goat polyclonal anti-PDX1 (Santa Cruz, dilution 1∶100), mouse monoclonal anti-AKT (Cell Signaling, dilution 1∶100), and rabbit polyclonal anti-caspase 3 cleaved (Cell Signaling, dilution 1∶100). To evaluate engraftment of human stem cells in the mouse pancreas we used rabbit polyclonal anti-human β2-microglobulin antibody at a dilution of 1∶100 (abcam). After washing with PBS, detection of bound primary antibodies was carried out with appropriate secondary antibodies conjugated with Alexa Fluor 488, or 568 (Invitrogen). To assess possible differentiation of human cells in the mouse pancreas, we used sequential steps for double staining. The slides were incubated first with the following antibodies: mouse monoclonal anti insulin (Sigma-Aldrich; dilution 1∶200) and mouse monoclonal anti-α-smooth muscle cell actin (Abcam; dilution 1∶100). Both antibodies cross-react with mouse and human insulin. Then slides were incubated with secondary antibodies conjugated with Alexa Fluor 568 donkey anti mouse at dilution 1∶200. After washing in PBS, the slides were incubated overnight at 4°C with anti-human β2-microglobulin antibody followed by Alexa Fluor 488 secondary antibody. Nuclear DNA was counterstained with 4′, 6-diamidino-2-phenylindole (DAPI, Vector Lab).

### B-cell Count

Briefly, the pancreatic sections were sequentially incubated with anti-insulin mouse monoclonal antibody, biotinylated rabbit anti-insulin antibody (Santa Cruz; dilution 1∶200), and streptavidin-alkaline phosphatase complex (Santa Cruz), for a period of 45 min each. The alkaline phosphatase activity was identified with new fuchsin under light microscopy. The sections were counterstained with Harris hematoxylin. The images of above stained sections were captured at 100 × magnification. We selected 3 sections separated from 200 µm and counted the number of insulin expressing cells in all 3 sections. We used immunofluorescent staining and confocal imaging to quantify β-cells expressing VEGF, human β2-microglobulin, and caspase 3 cleaved. We used pancreas from 3 mice from each group.

### Phase Contrast and Confocal Microscopy Analyses

Images were captured by digital camera connected with fluorescent microscope (Model Upright, Zeiss). Scanning confocal images for immunofluorescence analysis were obtained by a laser scanning confocal microscope (Leica Microsystems SP5, Mannheim, Germany).

### Real-time PCR Arrays

Pancreatic tissues were preserved with RNA Later (Invitrogen) at −20°C. Total RNA was extracted using RNeasy Minikit (Quiagen) according to the manufacturer’s instruction. The mRNAs from healthy control mice, STZ-induced diabetic mice and STZ-induced diabetic mice rescued by hBMSC-VEGF were obtained as described in manufacture’s manual. For the Real Time PCR array, all steps were performed according to the manufacturer’s protocol for the Roche Light Cycler 480. Briefly, RT2 First Strand Kit (SABiosciences, Frederick, USA) was used to convert mRNA to cDNA. This cDNA was then added to the SABiosciences RT2 SYBR Green qPCR Master Mix. Each sample was used to perform quantitative gene expression analysis on specific arrays for the insulin signaling pathway (Cat#PAMM-030-F). The online tool (http://www.sabiosciences.com/pcrarraydataanalysis.php) offered by the manufacturer was used to analyze the data including significant value and fold changes. Each set of data was repeated 4 times. Only significant results (p<0.05) were taken into consideration.

### DNA Extraction and Real-time PCR

Frozen tissues were homogenized, and genomic DNA was extracted using DNeasy Blood & Tissue Kit (Qiagen) from mouse organs and human BMSCs. Mouse DNA was isolated from identical tissues of non-transplanted NOD/SCID mice as used as negative control. In addition, human DNA was isolated from hBMSC cultures and used as positive control. Total DNA was assayed by UV absorbance. Real time-PCR was performed with 100 ng target DNA. To detect human DNA in the mouse tissues we used the previously reported human specific primers, targeting a unique and conserved region of human β-actin [26]. Endogenous mouse GAPDH gene (Qiagen, QT01658692) was also amplified as internal control. Real-Time PCR was carried out with iCycler (Bio-Rad), using QuantiFast SYBR Green PCR kit (Qiagen), following manufacture protocol. Absolute standard curves were obtained for the human β-actin and mouse GAPDH. To evaluate human specificity of human β-actin gene, standard curves were generated by serially diluting human genomic DNA in mouse DNA. Values are expressed in percent of human DNA infused as cells in mouse tissues. Each assay was carried on in triplicates and repeated at least 3 times.

### Statistical Analysis

All data were presented as mean ± SD and were compared by student t-test. The value of *p*<0.05 was considered to indicate statistical significance of the test results. Kaplan-Meier curves were used for the survival study and the log-rank (Maltel-Cox) test was used to determine statistical significance. For comparison of more than 2 groups we used one way ANOVA followed by Tukey test. For categorical data we used chi-square test.

## Results

### Human Bone Marrow Mesenchymal Stem Cells Expressing VEGF Ameliorated STZ-induced Hyperglycemia

Our hBMSCs expressed mesenchymal cell markers and differentiated into adipogenic and osteogenic cell lineages in vitro (data not shown), confirming their multipotent nature.

**Figure 1:**
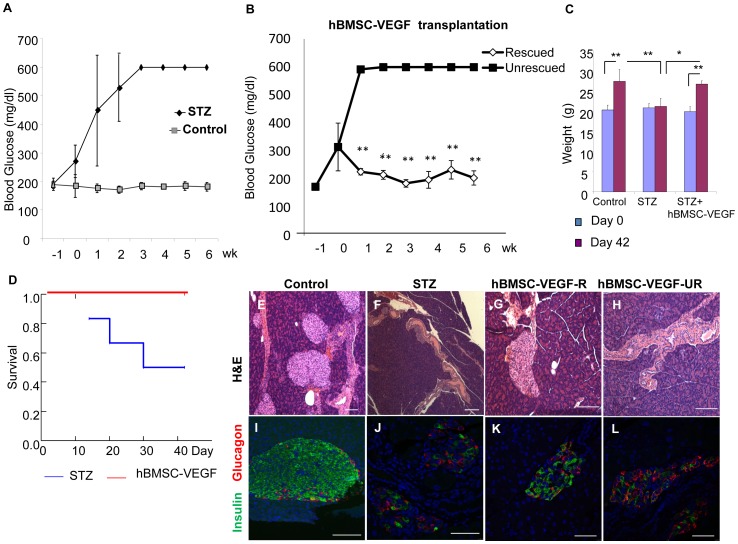
Human BMSCs-VEGF transplantation into chemically induced diabetic mice. Panel A shows blood glucose levels of control healthy mice (gray rectangle) versus STZ-induced diabetic mice (black diamond). Panel B shows blood glucose levels of mice rescued from diabetes after hBMSCs-VEGF treatment (Rescued, white diamond) versus unrescued mice (Unrescued, black rectangle). Panel C shows weights in gram (g) of control mice (control), diabetic mice (STZ) and diabetic mice treated with hBMSCs-VEGF (hBMSC-VEGF) indicating significant weight gain in control and hBMSCs-VEGF treated groups, compared with one in the diabetic group during 6 week study period (day 42; purple bar) from the moment of stem cell transplantation (Day 0; blue bar). Survival analysis (D) shows 100% survival of hBMSCs-VEGF treated mice (red line), compared with the significant reduction in diabetic (STZ) mice (blue line), *p<0.05*. Immunohistochemistry of pancreas by H&E staining (E,F,G,H) and immunofluorescence costaining (I,J,K,L) for insulin (green) and glucagon (red) shows various morphologies of pancreatic islets among different groups of mice, control healthy (E,I), STZ-induced diabetic (F,J), rescued (hBMSCs-VEGF-R; G,K) and unrescued mice (hBMSCs-VEGF-UR; H,L) by hBMSCs-VEGF treatment at the end of study period (6 weeks after cell injection). Scale bar: 50 µm. *p<0.01, **p<0.001.

Diabetes was induced in NOD/SCID mice with STZ, a cytotoxic agent that preferentially damages β-cells. All mice treated with STZ (n = 6) developed hyperglycemia 6–7 days after STZ injection and 50% of them died before 6 weeks (Fig. 1A). Control untreated mice (n = 5) maintained euglycemia during the study period (Fig. 1A). We tested the ability of hBMSCs genetically modified to transiently express VEGF gene to rescue diabetic mice. These cells were injected into the circulation of the STZ-induced diabetic mice (n = 9) one week after STZ treatment. Reversion of hyperglycemia was observed in 5 of 9 mice treated with hBMSCs-VEGF between 1 and 2 weeks after cell injection and near-normoglycemia remission was maintained for 6 weeks (Fig. 1B, ‘rescued’). Four of the mice treated with hBMSCs-VEGF failed to reverse hyperglycemia (‘unrescued’).

Although we divided into two subgroups, rescued and unrescued, all mice treated with hBMSCs-VEGF showed better clinical outcomes in terms of survival rate and weight gain compared with the STZ-induced diabetic mice (Fig. 1C,D). All of rescued and unrescued groups of mice gained weight significantly, which is comparable to the healthy control mice (Fig. 1C). These mice survived by the end time of the study in contrast to the high mortality rate of the diabetic mice (Fig. 1D, *p<0.05*). Overall, the response to treatment in terms of reversion of hyperglycemia was significantly higher in the mice treated with hBMSCs-VEGF compared with the mice receiving sham injection (*p = 0.025)*.

**Figure 2:**
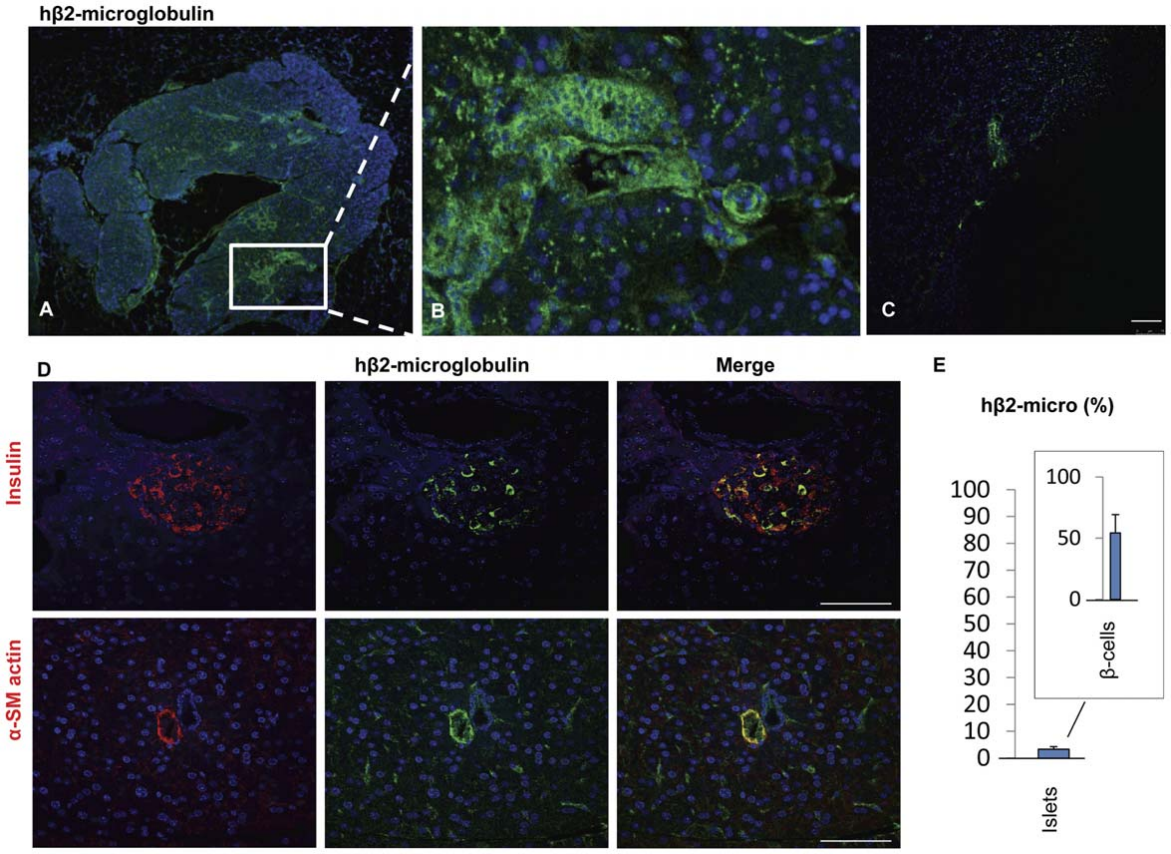
Engraftment of hBMSCs-VEGF in the injured pancreas. Immunofluorescence staining for human β-2-microglobulin (green) shows successful engraftment of hBMSCs-VEGF in the pancreas of rescued mice (A). Panel B shows higher magnification of inset in panel A indicating differentiation of human cells to vascular and ductal structures in the pancreas. However, in the pancreas of the unrescued mice (C) fluorescence immunostating for human β-2-microglobulin shows less human stem cell engraftment. Panel D shows a few cells in a pancreatic islet coexpressing human β-2-microglobulin (green) and insulin (red) in the upper row, and also a small vessel like structure inside the pancreas coexpressing human β-2-microglobulin (green) and α smooth muscle actin (red) in the bottom row. Scale bar: 50 µm. Panel E shows the percentage of the pancreatic islets expressing human β-2-microglobulin, and the percentage of β-cells expressing human β-2-microglobulin in the positive pancreatic islets (Inset) indicating 50% of engrafted hBMSCs-VEGF was differentiated into β-cells.

Histological examination at 6 weeks from transplantation showed severe alteration of the pancreatic islet morphology and significant reduction of the number of insulin- expressing cells in the STZ-induced diabetic mice (Fig. 1F,J) and in the unrescued mice (Fig. 1H,L). In contrast, the morphology of pancreatic islet was maintained and the staining pattern of insulin in the pancreatic islets of hBMSCs-VEGF treated mice (Fig. 1G,K) was very similar to one in the healthy control mice (Fig. 1E,I). Further investigation showed that hBMSCs-VEGF were robustly engrafted and diffusely survived in the pancreas (Fig. 2A–B), while fewer human cells were present in the pancreas of unrescued animals at 6 weeks after transplantation (Fig. 2C). Engraftment and survival of hBMSCs-VEGF in the mouse pancreas were also assessed by real-time PCR for a human-specific gene (Table 1). The pancreatic samples from mice treated with hBMSCs-VEGF showed variable amounts of human DNA. Small amounts of DNA were also present in the kidney of a few mice but not in other organs (Table 1).

**Table 1:** Human cell engraftment assayed by real-time PCR.

Animal/Cells	Pancreas	Kidney	Liver
1/hBMSC-VEGF	0.2±0.05	ND	NA
2/hBMSC-VEGF	0.18±0.07	ND	NA
3/hBMSC-VEGF	0.025±0.005	0.004±0.001	ND
4/hBMSC-VEGF	0.03±0.007	0.015±0.007	ND
1/hBMSC	0.008±0.0005	ND	NA
2/hBMSC	0.0048±0.001	ND	NA
3/hBMSC	ND	ND	ND
4/hBMSC	ND	ND	NA
1–3/no cells	ND	ND	NA

Percentage of human DNA infused as cells. NA, not assayed; ND, not detected. Mean±SD.

Furthermore hBMSCs-VEGF were able to differentiate into vessels and β-cells as confirmed by co-staining human β2-microglobulin which specifically stained human cells with either α-smooth muscle actin or insulin (Fig. 2D). Vascular differentiation was prominent inside the pancreas; however, the efficiency of differentiation into β-cells was low. It is interesting to note that only a small percentage of pancreatic islets were positive for human β2-microglobulin (Fig. 2E). However, those pancreatic islets containing human cells showed approximately half of the β-cells originated from human (Fig. 2E, right panel).

**Figure 3:**
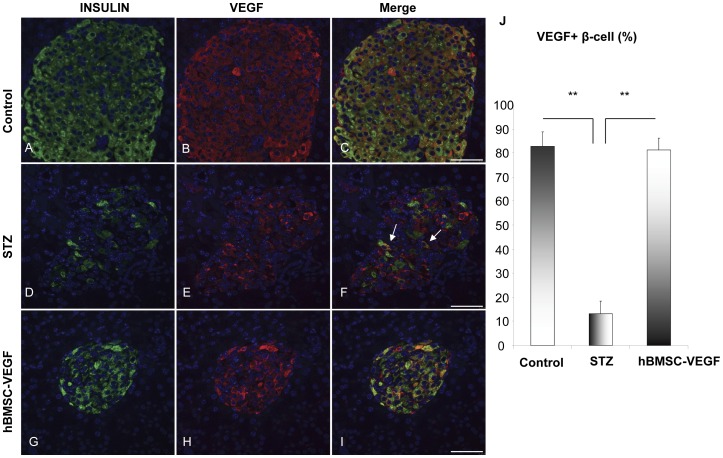
VEGF expression in the pancreatic islets. Immunocytochemistry shows different levels of coexpression of insulin (green) and VEGF (red) in the pancreatic islets of various groups of mice, control (A–C), diabetic (STZ;D–F), and rescued by hBMSCs-VEGF (G–I). Arrows in panel F show only few cells coexpressing insulin and VEGF in STZ treated group compared with other 2 groups. Panel J shows percentage of β-cells expressing VEGF in the pancreas of control mice similar to that of mice rescued by hBMSCs-VEGF that is significantly higher than that of diabetic mice (STZ). Scale bar: 50 µm. **p<0.001.

In the healthy age matched control mice, VEGF was uniquely expressed only in the pancreatic islets, mostly by the β-cells (Fig. 3A–C). After induction of diabetes with STZ, we observed a dramatic and significant reduction of VEGF expression in the β-cells (Fig. 3D–F), which was completely restored after treatment with hBMSCs-VEGF (Fig. 3A–J).

### Human Bone Marrow Mesenchymal Stem Cells Expressing PDX1 Transiently Ameliorated STZ-induced Hyperglycemia

**Figure 4:**
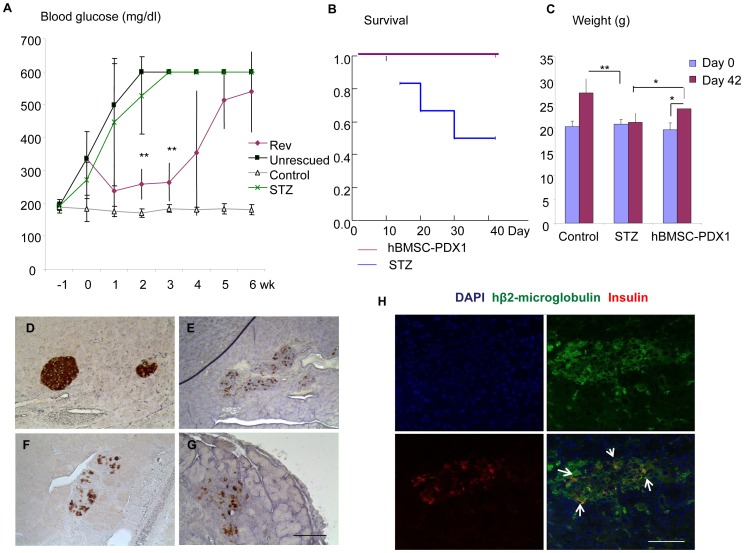
Human BMSCs-PDX1 transplantation in chemically induced diabetic mice. Panel A shows different patterns of blood glucose levels in various groups of mice, temporary reversed (purple diamond, Rev), unrescued (black rectangle, Unrescued), control (white triangle) and STZ-induced diabetic (green cross, STZ) during study time period. Survival analysis (B) of diabetic mice injected with hBMSCs-PDX1 (purple line) shows 100% survival compared with the significant lifespan reduction of diabetic mice (blue line, *p<0.05*) at 6 weeks. Panel C shows weights in gram (g) of control mice (control), diabetic mice (STZ) and diabetic mice treated with hBMSCs-PDX1, indicating significant weight gains in control and hBMSCs-PDX1treated groups compared with one in the diabetic group during 6 week study period (day 42; purple bar) from the moment of stem cell transplantation (Day 0; blue bar). Immunoperoxidase staining analysis in pancreases shows stronger expression of insulin in control healthy mice (D) compared with ones in other groups of mice, STZ-induced diabetic (E), temporary reversed (F), and unrescued (G). Panel H shows coexpression of human β-2-microglobulin (green) and insulin (red) in the injured pancreas suggesting de novo differentiation of β-cells from injected hBMSCs-PDX1. Nuclei were counterstained with DAPI (Blue). Scale bar: 50 µm. *p<0.05, **p<0.001.

We tried to determine if hBMSCs genetically modified to express PDX1 (hBMSCs-PDX1) were able to rescue diabetic mice. The cells were injected into the circulation of the diabetic mice (n = 8) 7 day after STZ treatment. Four mice showed reduction of the hyperglycemia in the following week after transplantation (‘temporary reversed’), while the remained 4 mice maintained severe hyperglycemia (‘unrescued’, Fig. 4A). Interestingly, the temporary reversed mice maintained near-normoglycemic remission for 2–3 weeks, and then developed severe hyperglycemia again. All mice (‘temporary reversed’ and ‘unrescued’) survived at 6 weeks from transplantation, compared with the significant drop in survival rate of the diabetic mice (Fig. 4B, *p<0.05*), and gained significant weight compared with the diabetic control mice (Fig. 4C), indicating a better clinical outcome. Isolated pancreas from ‘temporary reversed’ as well as ‘unrescued’ mice analyzed for immunostaining against insulin showed reduction of insulin expression in the pancreatic islets compared (Fig. 4F,G) with healthy control mice (Fig. 4D) and similar to the STZ-induced diabetic mice (Fig. 4E). Staining for human beta-2 microglobulin clearly showed the engraftment of human cells in pancreases (Fig. 4H). In both groups (’temporary reversed’ and ‘unrescued’), we found none of vessel-like structures were differentiated from the hBMSCs-PDX1. A few transplanted cells expressing insulin in islet structures were noted as shown in Fig. 4H, implicating functional differentiation into β-cells.

### Human Mesenchymal Stem Cell from Bone Marrow Alone did not Induce Recovery of STZ-Induced Diabetes

**Figure 5:**
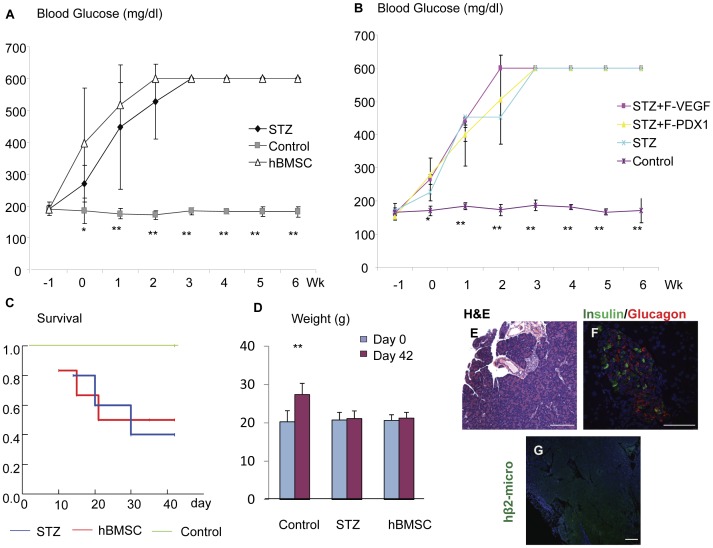
Human BMSC transplantation in chemical induced diabetic mice. Panel A shows blood glucose levels in 3 groups of mice, STZ-induced diabetic (black diamond, STZ), treated with hBMSCs (white triangle, hBMSC), and healthy control mice (gray rectangle) during 6 week period. In panel B only healthy control mice (n = 4; purple cross) maintain normoglycemia while other groups [STZ-induced diabetic (STZ; n = 4, blue cross), STZ-induced diabetic mice after treatment with fibroblast expressing VEGF (F-VEGF; n = 4; purple square) and fibroblast expressing PDX1 (F-PDX1; n = 4; yellow triangle)] develop severe hyperglycemia during 6 week study period. Survival analysis graph (C) shows lifespan reductions in diabetic mice injected with hBMSCs (red line) and non injected diabetic mice (STZ, blue line) compared with one of control mice (*p<0.05*). Panel D shows only healthy control mice continue to gain weight compared with diabetic mice treated with hBMSCs and untreated diabetic mice. Immunohistochemistry of pancreases from the diabetic mice treated with hBMSCs (E–G); H&E staining shows reduced size of pancreatic islets and altered morphology (E); fluorescence staining for insulin (green) and glucagon (red) shows low level of insulin expression (F); fluorescence immunostaining for human β2-microglobulin (green) shows very poor engraftment of the hBMSCs (G). Scale bar: 50 µm. *p<0.05, **p<0.001.

In contrast with two above results, hBMSCs without genetic modification were not able to ameliorate diabetic phenotypes. Diabetic mice (n = 6) treated with one intra-left ventricular injection of 1×10^6^ hBMSCs at day 7 from STZ injection continued to maintain severe hyperglycemia (Fig. 5A). Control experiments were carried on with fibroblasts expressing VEGF or PDX1 and did not show improvement of hyperglycemia after fibroblast transplantation (Fig. 5B). More than 50% of mice treated with hBMSCs died before 6 weeks showing a survival rate similar to the STZ-induced diabetic mice and significantly lower than the healthy control mice (Fig. 5C, *p<0.05*). In addition, survived mice failed to gain weight (Fig. 5D) at 6 weeks post-transplantation. Histological examination showed severe alteration of the pancreatic islet morphology in this group (Fig. 5E) similar to the STZ-induced diabetic mice. The pancreatic islets of mice receiving hBMSCs showed reduction in insulin expression with the characteristic inversion in the ratio of insulin/glucagon cells (Fig. 5F). Further investigation showed poor engraftment of hBMSCs in the pancreas at 6 weeks after transplantation (Fig. 5G). Detection of human DNA in the mouse pancreas at 6 weeks post-transplantation was low (Table 1). We were able to detect human DNA in two out of four tested mice with a lower concentration compared with the mice treated with hBMSCs-VEGF, confirming a lower engraftment and/or survival of donor cell in the recipient pancreas.

### Contribution of Genetically Modified Human Bone Marrow Mesenchymal Stem Cells to β Cell Recovery: Endogenous Versus Transplant-derived β-cell Differentiation

**Figure 6:**
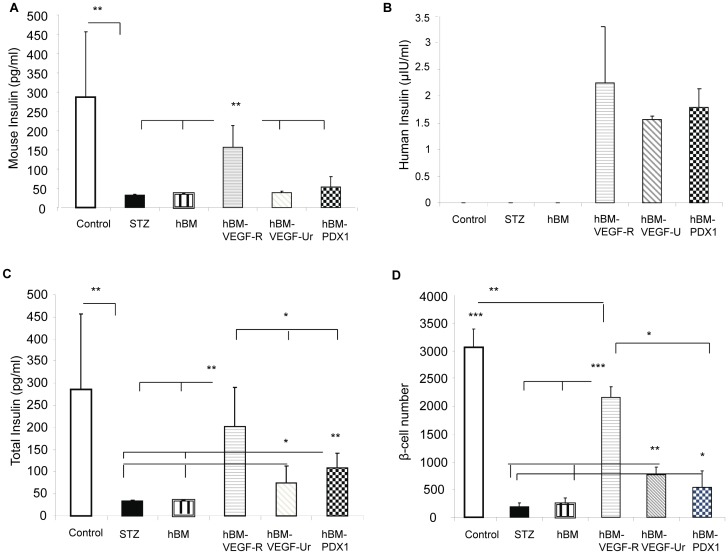
Serum insulin levels and β-cell number. Panel A shows only 2 groups of mice, healthy control and rescued by hBMSCs-VEGF (hBM-VEGF-R) have significantly higher mouse insulin levels compared with other groups of mice, diabetic (STZ), treated with hBMSCs (hBM), unrescued by hBMSCs-VEGF (hBM-VEGF-Ur), and treated with hBMSCs-PDX1 (hBM-PDX1). There is no significant difference of mouse insulin level between healthy control mice and diabetic mice rescued by hBMSCs-VEGF (hBM-VEGF-R). Panel B shows human insulin levels in various groups of mice, 3 mice rescued by hBMSCs-VEGF, 2 mice unrescued by hBMSCs-VEGF, and 5 mice treated with hBMSCs-PDX1 (B) indicating functional de novo differentiation of β-cells. Panel C shows diabetic mice rescued by hBMSCs-VEGF (hBM-VEGF-R) have significantly higher total insulin level (human and mouse insulin) than other groups [diabetic mice (STZ), diabetic mice treated with hBMSCs (hBM), diabetic mice unrescued by hBMSCs-VEGF (hBM-VEGF-Ur), and diabetic mice treated with hBMSCs-PDX1 (hBM-PDX1)], which is not significantly different from healthy control mice. Mice unrescued by hBMSCs-VEGF (hBM-VEGF-Ur) and treated with hBMSCs-PDX1 (hBM-PDX1) have significantly higher total insulin levels than diabetic mice and diabetic mice treated with hBMSCs, but lower than control mice and mice rescued by hBMSCs-VEGF (hBM-VEGF-R). β-cell number is higher in the healthy control mice compared with ones in other groups (D). Mice rescued by hBMSCs-VEGF have significantly higher β-cell number than other groups of mice (diabetic, treated with hBMSCs, unrescued by hBMSCs-VEGF, and treated with hBMSCs-PDX1), which is still significantly lower than healthy control mice. Diabetic mice unrescued by hBMSCs-VEGF and treated with hBMSCs-PDX1 have significantly higher β-cell number than diabetic mice and diabetic mice treated with hBMSCs, but significantly lower than mice rescued by hBMSCs-VEGF. *p<0.05, **p<0.01, ***p<0.001.

To evaluate the possible contribution of hBMSCs-VEGF to the recovery of β-cells and whether the reversion of diabetes was secondary to a direct differentiation of hBMSCs to β-cells or secondary to endogenous β-cell regeneration, we measured both mouse and human serum insulin. Only the mice rescued by hBMSCs-VEGF had significantly higher level of mouse insulin compared with diabetic mice and diabetic mice treated with hBMSCs or hBMSCs-PDX1 (Fig. 6A). Interestingly, more than half of the mice treated with hBMSCs-VEGF and hBMSCs-PDX1 (5 out of 9 and 5 out of 8, respectively) showed low but detectable levels of human insulin, demonstrating de novo differentiation of human BMSCs into functional β-cells (Fig. 6B). The levels of total serum insulin were significantly higher in the mice treated with hBMSCs-VEGF and hBMSCs-PDX1 compared with the diabetic mice and the diabetic mice treated with hBMSCs (Fig. 6C). The result showed clear correlation between the level of insulin and the number of β-cells, which was again higher in the mice treated with hBMSCs-VEGF and hBMSCs-PDX1 than two other groups (Fig. 6D). In addition, even unrescued groups of mice from hBMSCs-VEGF and hBMSCs-PDX1 with persistent hyperglycemia showed significantly higher levels of total serum insulin and number of endogenous β-cells compared with other groups resulting in overall better clinical outcomes. More importantly, the group of mice with sustained near-normoglycemia remission, treated with hBMSCs-VEGF, had the highest level of mouse insulin and β-cell number among groups, suggesting that sustained reversion of diabetes was secondary to endogenous β-cell regeneration or recovery rather than transplant-derived β-cell differentiation.

### Endogenous β-cell Recovery in Mouse Rescued by hBMSCs-VEGF was Secondary to Activation of Insulin/IGF Receptor Signaling Pathway

**Figure 7:**
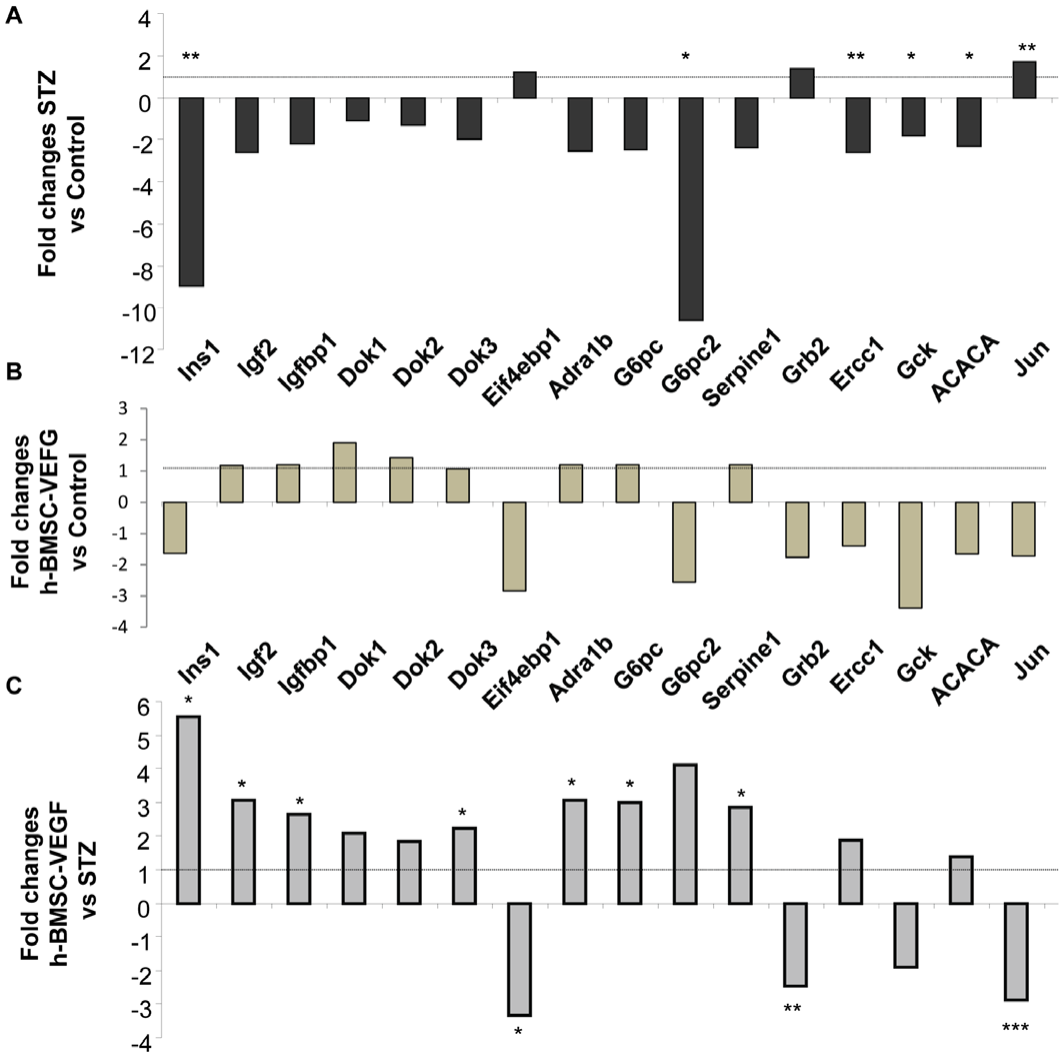
Insulin/IGF1 receptor signaling pathway in the pancreas of diabetic mice, healthy control mice and mice rescued by hBMSCs-VEGF: PCR array profile. Figure 7 shows fold changes of various gene expression in diabetic mice (STZ) compared with ones in healthy control (A); mice rescued by hBMSCs-VEGF compared with healthy control mice (B); mice rescued by hBMSCs-VEGF compared with diabetic mice (C). *p<0.05, **p<0.01, ***p<0.001.

To evaluate the possible mechanism of endogenous β-cell recovery in the rescued mice treated with hBMSCs-VEGF we compared PCR array data of the pancreases from the healthy and diabetic age matched control mice. We noted a clear trend of decreasing expression of mouse genes related with insulin receptor signaling pathway in pancreases of the diabetic mice compared with the healthy control (Fig. 7A). In the STZ-induced diabetic mice, there was a significant decrease in expression of Insulin1, as expected. In addition, a significant decrease in gene expression of excision repair cross-complementing rodent repair deficiency 1 (Ercc1), glucokinase (Gck), and acetyl-coenzyme A carboxylase alpha (ACACA) with an increased expression of jun. However, mice rescued by hBMSCs-VEGF showed a similar PCR-array profile as seen in the healthy control mice without significant changes in gene expression (Fig. 7B). Interestingly, PCR array result from the rescued mice showed the significant up-regulation of the mouse genes involved in the insulin/IGF signaling pathway compared with one from the diabetic mice (Fig. 7C). In particular, insulin was significantly upregulated in the rescued mice while it was downregulated in STZ-induced diabetes mice, which is comparable to one in healthy control group. Insulin receptor associate proteins such as insulin growth factor 2 (IGF2), insulin growth factor binding protein 1(Igfbp1), and Dok3 were significantly upregulated in rescued mice. All target genes for phosphatidylinositol 3-kinase (PI-3K) pathway such as adrenergic receptor alpha1d (Adra1d), glucose-6-phosphatase catalytic (G6pc), glucose-6-phosphatase catalytic 2 (G6pc2), and serpine 1 were also upregulated while eukaryotic translational initiation factor 2B, subunit 1 (Eif4ebp1), growth factor receptor bound protein 2 (Grb2) and jun were significantly downregulated in the rescued mice as compared with ones in the diabetic mice (Fig. 7C).

**Figure 8:**
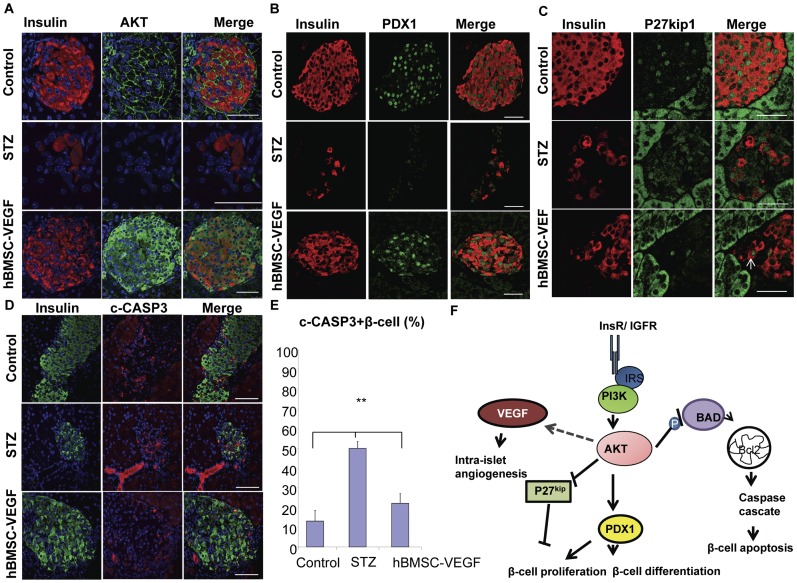
Insulin/IGF1 receptor/PI-3K/AKT pathway in the pancreas of diabetic mice, healthy control mice, and mice rescued by hBMSCs-VEGF. Immunostaining of pancreatic sections from control healthy mice (control), diabetic mice (STZ), and diabetic mice rescued by hBMSCs-VEGF (hBMSC-VEGF) for AKT (green), insulin (red) and merge in yellow (A); PDX1 (green) and insulin (red) (B); p27-kip1 (green) and insulin (red) (C); Caspase 3 cleaved (red), insulin (green), and merge in yellow (D). Only few nuclei in pancreatic islets of rescued mice were positive for p27-kip1 (arrow in panel C). Nuclei were counterstained with DAPI (blue). Panel E shows diabetic mice (STZ) has significantly higher percentage of β-cells expressing Caspase 3 cleaved (c-CASP3) in the pancreas than control mice (control) and diabetic mice rescued by hBMSCs-VEGF. In panel F, we present schematic summary of the Insulin receptor/PI-3K/AKT signaling pathway involving endogenous β-cell regeneration mediated by treatment with hBMSCs-VEGF: reduced β-cell apoptosis, increased β-cell differentiation and proliferation through PDX1 expression and down regulation of the cell cycle inhibitor p27-kip, and improved intra-islet angiogenesis through regulation of VEGF expression (F). Scale bar: 50 µm. ** p<0.01.

To further explore the molecular mechanism for the reversion of diabetes and β-cell recovery/regeneration in diabetic mice treated with hBMSCs-VEGF, pancreatic islets from control healthy mice, STZ-induced diabetic mice, and diabetic mice rescued by hBMSCs-VEGF were examined by high resolution confocal microscopy imaging system to assess Insulin/IGF receptor/PI3-K downstream proteins. AKT protein was highly expressed in the pancreatic islets of healthy mice mostly on the plasma membranes (Fig. 8A, upper panel) while dramatically reduced in diabetic condition (Fig. 8A, intermediate panel). Interestingly, after injection of hBMSCs-VEGF, pancreatic islets of rescued mice showed upregulation of AKT expression with distribution not only in the plasma membrane but also strongly in the cytoplasm (Fig. 8A, lower panel). Activation of Insulin/IGF receptor/PI-3K/AKT pathway is associated with increase of β-cell mass through activation of downstream proteins required for β-cell proliferation, differentiation and survival, such as PDX1 [27], [28] and P27Kip1 [29], [30]. Associated with preservation and/or regeneration of β-cells and insulin secretion, mice rescued by hBMSCs-VEGF showed a strong nuclear localization of PDX1 (Fig. 8B, lower panel) similar to the control healthy mice (Fig. 8B, upper panel). In contrast PDX1 was less detected in β-cell nuclei of the diabetic mice (Fig. 8B, intermediate panel) with a staining that appears to be weaker. In addition, virtually all β-cell nuclei of the control mice were positive for p27^Kip1^ (Fig. 8C, upper panel), a cell cycle inhibitor protein negatively regulated through the PI-3K/AKT pathway. This is consistent with the slow β-cell replication in postnatal life [31]. After induction of diabetes most β-cell nuclei were positive for p27^Kip1^ (Fig. 8C, intermediate panel), suggesting the persistent inhibition of β-cell replication. In contrast confocal microscopy analysis showed the remarkable decrease of p27^Kip1^ protein expression in the pancreatic islets of mice rescued by hBMSCs-VEGF, with only isolated positive nuclei (Fig. 8C, lower panel), strongly indicating activation of β-cell replication.

We next measured caspase 3 cleaved (c-CASP3) to assess β-cell apoptosis in relation with activation of the Insulin receptor/PI-3K/AKT pathway. Pancreatic islets from diabetic mice showed dramatic increase in c-CASP3 compared with ones from control and rescued mice (Fig. 8D). Compared with the diabetic mice, the rescued mice showed significantly lower number of c-CASP3-positive β-cells, similar to the healthy control mice (Fig. 8E). This is consistent with anti-apoptotic signal mediated by activation of PI-3K pathway (Fig. 8F).

## Discussion

Stem cell therapy may be a desirable alternative to pancreas and pancreatic islet transplantation, and stem cells from bone marrow represent an attractive source. Animal studies suggested the possible ameliorate of diabetes by bone marrow stem cell therapy [3], [7], [8], [9], [10], [11]. However, the mechanism related to β-cell recovery required elucidation. Here, for the first time we tested the hypothesis of de novo β-cell differentiation from hBMSCs versus endogenous β-cell regeneration mediated by hBMSCs, using the transient expression of PDX1 and VEGF.

In contrast to previous reports [3], [7], [8], [9], [10], [11] hBMSCs alone were not able to reverse hyperglycemia in our animal model. This can be attributed to various differences in stem cell populations, mouse strains, mouse models, and experimental designs among research groups. Some studies used hematopoietic stem cells from bone marrow [3], [32] or mouse mesenchymal bone marrow stem cells [8], [9], [10], [11]. In addition C57BL/6 mice were used for STZ-induced diabetes model [8], [10], [11], which can explain different outcomes in response to STZ, degree of diabetes, blood glucose levels and responses to treatment. Only one previous report [7] is the closest to our study: same type of stem cells, same cell delivery method (intracardiac injection) and same mouse strain. However in this report the dosage of STZ used was adjusted to produce nonlethal hyperglycemia and the improvement in blood glucose control was achievable only with multiple injections of human cells. The higher glucose level and mortality of our mice in addition to a single stem cell injection can explain obtaining different results.

We achieved sustained recovery from diabetes following injection of hBMSCs overexpressing VEGF. We observed an efficient engraftment of hBMSCs-VEGF in the pancreas of the diabetic mice, and its successful differentiation into blood vessels and to lesser degree into β-cells. For the first time, we reported here detectable levels of human insulin confirming a successful chimerism in the mouse. However, the sustained near- normoglycemia remission was not fully supported by the low level of human insulin, and the low number of β-cells from hBMSCs-VEGF. The significantly higher level of mouse insulin in rescued mice suggested that the endogenous β-cell regeneration is the predominant mechanism behind the sustained clinical recovery. Taken together, the de novo intra pancreatic angiogenesis from hBMSCs-VEGF along with the endogenous activation of the insulin/IGF receptor signaling pathway strongly support regeneration and functional recovery of endogenous β-cells.

To highlight this concept, we performed a parallel experiment using hBMSCs expressing PDX1. The previous report showed the possible direct differentiation of human hBMSCs into β-cells *in vitro* after transfection with a virus vector encoding PDX1 [17], supporting the possible role of PDX1 to direct differentiation of hBMSCs into insulin-producing cells in our *in vivo* model. Interestingly, human BMSCs-PDX1 could differentiate to β-cells in the diabetic pancreas with approximately the same efficiency of hBMSCs-VEGF confirmed by similar detectable levels of serum human insulin and by immunohistochemistry. However, transplantation of hBMSCs-PDX1 into diabetic mice resulted in only transient recovery. The overall efficiency of hBMSCs-PDX1 engraftment and their differentiation into blood vessels were significantly lower than those obtained from hBMSCs-VEGF, which is correlated with the disparate clinical outcomes. We anticipate that even engrafted hBMSCs-PDX1 could not survive and/or maintain a normal function for long time due to lack of necessary supplies from host environment (damaged pancreas). VEGF-A has been known to play a key role in maintaining normal intra-islet vascularization [33], [34]. In addition, VEGF is known to enhance proliferation, survival and differentiation of bone marrow mesenchymal stem cells [35]. Overexpression of VEGF in BMSCs increased revascularization and myocardial recovery after injury [36], and neutralizing anti-VEGF antibodies inhibited the BMSC-initiated angiogenic response *in vivo* [37]. Moreover, the β-cell-specific VEGF-A deficient mouse showed the altered insulin secretion despite maintaining normal β-cell mass [38] and the lost of capacity to induce expansion of β-cell mass after STZ-induced diabetes followed by bone marrow transplantation compared with the wild-type mouse [39]. It has been reported that BMSCs improved revascularization and function of pancreatic islets after transplantation [40]. Bone marrow mesenchymal stem cells can not only promote endogenous angiogenesis [41], but directly differentiate into smooth muscle [42] and endothelial cell phenotypes [43] *in vitro,* and into functional vascular structures [44], [45] and contribute to myocardial recovery after injury *in vivo* [46].

The pancreas of mice rescued by hBMSCs-VEGF showed upregulation of insulin receptor associated gene, such as Ins1, Igf2, Igfbp1 as well as Dok1, 2 and 3. Recent extensive studies have shown the importance of insulin regulating β-cell function [47]. Our results showed that Insulin/IGF receptor coupling with insulin receptor soluble (IRS) proteins activated the downstream effector pathway PI-3K. Several gene targets in the PI-3K pathway were upregulated including Adra1d, G6pc, G6pc2, and Serpine 1 in the rescued group. In contrast, Grb2, generally thought to affect Ras and mitogen-activated protein kinase signaling, was significantly downregulated in the rescued mice. Consistent with a previous report [48], the expression of Jun was increased in the diabetic pancreas while it was significantly downregulated in the rescued mice. Insulin/IGF receptor/PI-3K signaling mediates several pathways related with proliferation and anti-apoptosis in most mammalian cells including pancreatic islets [49]. AKT is a critical mediator of the Insulin/IGF receptor/PI-3K pathway and overexpression of active AKT1 in β-cells significantly increased β-cell size and total islet mass [50]. We showed a significant decrease of AKT expression in the pancreatic islets of diabetic mice, compared with control and rescued mice. Interestingly, pattern of AKT distribution was mostly on the cell membrane of the β-cells of the healthy control mice, while it was highly expressed in both cell membrane and cytoplasm of the β-cells in the rescued mice. It is well known that AKT activation takes place on the cell membrane [51]. On the other hand, it has been reported that translocation of AKT in the cytoplasm and nucleus after stimulation with growth factors such us insulin and IGF1 could mediate potential anti-apoptotic mechanisms [52], [53]. Activation of Insulin/IGF signaling through PI-3K/ATK pathway could induce reduction of apoptosis by cytoplasmic sequestration of BAD that prevented BCL2 activation and subsequently caspase activation [54]. Thus our data showed increased apoptosis in the diabetic mouse pancreas measured by the increased number of β-cells expressing caspase 3 cleaved. Accordingly diabetic mice rescued by hBMSCs-VEGF showed a significant reduction in apoptosis.

In keeping with the observation of enhanced Insulin receptor/PI-3K/AKT signaling, the pancreatic islets of the rescued mice showed greater expression and nuclear localization of PDX1 compared with diabetic mice. PDX1 is a well known downstream transcriptional target of insulin signaling and it is required for β-cell growth and differentiation [27]. Moreover the expression level of p27^Kip1^, a cell cycle inhibitor known to be negatively regulated by PI-3K/AKT pathway in β-cells through FoxO1 [31] and Gsk-3β [30], was significantly reduced in the rescued group while up-regulated in control and diabetic pancreatic islets. This finding confirmed the increased proliferative signal in the rescued pancreatic islets through the Insulin receptor/PI-3K/ATK pathway, compared with ones in both control healthy mice and diabetic mice.

In addition, PI-3K signaling is also known to modulate VEGF expression in the endothelial cells and to induce angiogenesis [29] through v-Src [55]. Moreover, VEGF expression is predominant in the pancreatic β-cells and VEGF receptor 2 (VEGFR2) is highly expressed in the intra-islet capillary [20]. Our data showed a dramatic increase in VEGF expression in the β-cells of rescued mice compared with diabetic mice, implying the possible activation of VEGF expression via PI-3K/AKT pathway.

Taken together, our results suggest that hBMSCs-VEGF induce reversion of diabetes mainly by induction of endogenous β-cell regeneration through the generation of a favorable microenvironment mediated by the activation of the Insulin/IGF1 receptor/PI-3K/AKT pathway. Activation of this pathway in the β-cells improves cell survival through inhibition of apoptosis,s and induces β-cell differentiation and proliferation through activation of PDX1 expression and inhibition of P27^Kip1^_._ In addition, we provide an evidence of a possible new mechanism of β-cell recovery/regeneration through modulation of intra-islet angiogenesis. The activation of the Insulin/IGF signaling through the PI-3K pathway in the diabetic mice rescued by hBMSCs-VEGF induces VEGF expression in the β-cells, correlated with β-cell recovery (Fig. 8F).

In conclusion, our work provides new insight into the mechanism of β-cell recovery after injury mediated by hBMSC therapy and demonstrates the possible clinical benefit of hBMSCs expressing VEGF for the treatment of insulin-dependent diabetes.

## References

[1] Ianus A, Holz GG, Theise ND, Hussain MA (2003) In vivo derivation of glucose-competent pancreatic endocrine cells from bone marrow without evidence of cell fusion. J Clin Invest 111: 843–850. 12639990 10.1172/JCI16502 PMC153767

[2] Zhao M, Amiel SA, Ajami S, Jiang J, Rela M, et al (2008) Amelioration of streptozotocin-induced diabetes in mice with cells derived from human marrow stromal cells. PLoS One 3: e2666. 18628974 10.1371/journal.pone.0002666 PMC2441861

[3] Hess D, Li L, Martin M, Sakano S, Hill D, et al (2003) Bone marrow-derived stem cells initiate pancreatic regeneration. Nat Biotechnol 21: 763–770. 12819790 10.1038/nbt841

[4] Alvarez SS, Jimenez LM, Murillo AZ, Gomez IG, Ligero JM, et al (2006) A new approach for bone marrow-derived stem cells intrapancreatic autotransplantation in diabetic rats. Microsurgery 26: 539–542. 17006956 10.1002/micr.20283

[5] Banerjee M, Kumar A, Bhonde RR (2005) Reversal of experimental diabetes by multiple bone marrow transplantation. Biochem Biophys Res Commun 328: 318–325. 15670786 10.1016/j.bbrc.2004.12.176

[6] ksadHasegawa Y, Ogihara T, Yamada T, Ishigaki Y, Imai J, et al (2007) Bone marrow (BM) transplantation promotes beta-cell regeneration after acute injury through BM cell mobilization. Endocrinology 148: 2006–2015. 17255204 10.1210/en.2006-1351

[7] Lee RH, Seo MJ, Reger RL, Spees JL, Pulin AA, et al (2006) Multipotent stromal cells from human marrow home to and promote repair of pancreatic islets and renal glomeruli in diabetic NOD/scid mice. Proc Natl Acad Sci U S A 103: 17438–17443. 17088535 10.1073/pnas.0608249103 PMC1634835

[8] Urban VS, Kiss J, Kovacs J, Gocza E, Vas V, et al (2008) Mesenchymal stem cells cooperate with bone marrow cells in therapy of diabetes. Stem Cells 26: 244–253. 17932424 10.1634/stemcells.2007-0267

[9] Fiorina P, Jurewicz M, Augello A, Vergani A, Dada S, et al (2009) Immunomodulatory function of bone marrow-derived mesenchymal stem cells in experimental autoimmune type 1 diabetes. J Immunol 183: 993–1004. 19561093 10.4049/jimmunol.0900803 PMC3895445

[10] Ezquer FE, Ezquer ME, Parrau DB, Carpio D, Yanez AJ, et al (2008) Systemic administration of multipotent mesenchymal stromal cells reverts hyperglycemia and prevents nephropathy in type 1 diabetic mice. Biol Blood Marrow Transplant 14: 631–640. 18489988 10.1016/j.bbmt.2008.01.006

[11] Ezquer F, Ezquer M, Simon V, Conget P (2011) The antidiabetic effect of MSCs is not impaired by insulin prophylaxis and is not improved by a second dose of cells. PLoS One 6: e16566. 21304603 10.1371/journal.pone.0016566 PMC3029393

[12] Milanesi A, Lee JW, Xu Q, Perin L, Yu JS (2011) Differentiation of nestin-positive cells derived from bone marrow into pancreatic endocrine and ductal cells in vitro. J Endocrinol 209: 193–201. 21330336 10.1530/JOE-10-0344

[13] Kabos P, Ehtesham M, Kabosova A, Black KL, Yu JS (2002) Generation of neural progenitor cells from whole adult bone marrow. Exp Neurol 178: 288–293. 12504887 10.1006/exnr.2002.8039

[14] Zeng Z, Yuan X, Liu G, Zeng X, Ng H, et al (2007) Manipulation of proliferation and differentiation of human bone marrow-derived neural stem cells in vitro and in vivo. J Neurosci Res 85: 310–320. 17131390 10.1002/jnr.21131

[15] Tang DQ, Cao LZ, Burkhardt BR, Xia CQ, Litherland SA, et al (2004) In vivo and in vitro characterization of insulin-producing cells obtained from murine bone marrow. Diabetes 53: 1721–1732. 15220196 10.2337/diabetes.53.7.1721 PMC3422216

[16] Chen LB, Jiang XB, Yang L (2004) Differentiation of rat marrow mesenchymal stem cells into pancreatic islet beta-cells. World J Gastroenterol 10: 3016–3020. 15378785 10.3748/wjg.v10.i20.3016 PMC4576264

[17] Karnieli O, Izhar-Prato Y, Bulvik S, Efrat S (2007) Generation of insulin-producing cells from human bone marrow mesenchymal stem cells by genetic manipulation. Stem Cells 25: 2837–2844. 17615265 10.1634/stemcells.2007-0164

[18] Fujitani Y, Fujitani S, Boyer DF, Gannon M, Kawaguchi Y, et al (2006) Targeted deletion of a cis-regulatory region reveals differential gene dosage requirements for Pdx1 in foregut organ differentiation and pancreas formation. Genes Dev 20: 253–266. 16418487 10.1101/gad.1360106 PMC1356115

[19] Holland AM, Gonez LJ, Naselli G, Macdonald RJ, Harrison LC (2005) Conditional expression demonstrates the role of the homeodomain transcription factor Pdx1 in maintenance and regeneration of beta-cells in the adult pancreas. Diabetes 54: 2586–2595. 16123346 10.2337/diabetes.54.9.2586

[20] Brissova M, Shostak A, Shiota M, Wiebe PO, Poffenberger G, et al (2006) Pancreatic islet production of vascular endothelial growth factor–a is essential for islet vascularization, revascularization, and function. Diabetes 55: 2974–2985. 17065333 10.2337/db06-0690

[21] Nikolova G, Jabs N, Konstantinova I, Domogatskaya A, Tryggvason K, et al (2006) The vascular basement membrane: a niche for insulin gene expression and Beta cell proliferation. Dev Cell 10: 397–405. 16516842 10.1016/j.devcel.2006.01.015

[22] Nicholson JM, Arany EJ, Hill DJ (2010) Changes in islet microvasculature following streptozotocin-induced beta-cell loss and subsequent replacement in the neonatal rat. Exp Biol Med (Maywood) 235: 189–198. 20404034 10.1258/ebm.2009.009316

[23] Christofori G, Naik P, Hanahan D (1995) Vascular endothelial growth factor and its receptors, flt-1 and flk-1, are expressed in normal pancreatic islets and throughout islet cell tumorigenesis. Mol Endocrinol 9: 1760–1770. 8614412 10.1210/mend.9.12.8614412

[24] Chae HY, Lee BW, Oh SH, Ahn YR, Chung JH, et al (2005) Effective glycemic control achieved by transplanting non-viral cationic liposome-mediated VEGF-transfected islets in streptozotocin-induced diabetic mice. Exp Mol Med 37: 513–523. 16391512 10.1038/emm.2005.64

[25] Narang AS, Sabek O, Gaber AO, Mahato RI (2006) Co-expression of vascular endothelial growth factor and interleukin-1 receptor antagonist improves human islet survival and function. Pharm Res 23: 1970–1982. 16906455 10.1007/s11095-006-9065-7

[26] Malek A, Catapano CV, Czubayko F, Aigner A (2010) A sensitive polymerase chain reaction-based method for detection and quantification of metastasis in human xenograft mouse models. Clin Exp Metastasis 27: 261–271. 20364399 10.1007/s10585-010-9324-1

[27] Kulkarni RN, Jhala US, Winnay JN, Krajewski S, Montminy M, et al (2004) PDX-1 haploinsufficiency limits the compensatory islet hyperplasia that occurs in response to insulin resistance. J Clin Invest 114: 828–836. 15372107 10.1172/JCI21845 PMC516265

[28] Kitamura T, Nakae J, Kitamura Y, Kido Y, Biggs WH, 3rd, et al (2002) The forkhead transcription factor Foxo1 links insulin signaling to Pdx1 regulation of pancreatic beta cell growth. J Clin Invest 110: 1839–1847. 12488434 10.1172/JCI200216857 PMC151657

[29] Jiang BH, Zheng JZ, Aoki M, Vogt PK (2000) Phosphatidylinositol 3-kinase signaling mediates angiogenesis and expression of vascular endothelial growth factor in endothelial cells. Proc Natl Acad Sci U S A 97: 1749–1753. 10677529 10.1073/pnas.040560897 PMC26507

[30] Tanabe K, Liu Z, Patel S, Doble BW, Li L, et al (2008) Genetic deficiency of glycogen synthase kinase-3beta corrects diabetes in mouse models of insulin resistance. PLoS Biol 6: e37. 18288891 10.1371/journal.pbio.0060037 PMC2245985

[31] Folli F, Okada T, Perego C, Gunton J, Liew CW, et al (2011) Altered insulin receptor signalling and beta-cell cycle dynamics in type 2 diabetes mellitus. PLoS One 6: e28050. 22140505 10.1371/journal.pone.0028050 PMC3227614

[32] Hasegawa Y, Ogihara T, Yamada T, Ishigaki Y, Imai J, et al (2007) Bone marrow (BM) transplantation promotes beta-cell regeneration after acute injury through BM cell mobilization. Endocrinology 148: 2006–2015. 17255204 10.1210/en.2006-1351

[33] Esser S, Wolburg K, Wolburg H, Breier G, Kurzchalia T, et al (1998) Vascular endothelial growth factor induces endothelial fenestrations in vitro. J Cell Biol 140: 947–959. 9472045 10.1083/jcb.140.4.947 PMC2141756

[34] Issbrucker K, Marti HH, Hippenstiel S, Springmann G, Voswinckel R, et al (2003) p38 MAP kinase–a molecular switch between VEGF-induced angiogenesis and vascular hyperpermeability. FASEB J 17: 262–264. 12490545 10.1096/fj.02-0329fje

[35] Lin H, Shabbir A, Molnar M, Yang J, Marion S, et al (2008) Adenoviral expression of vascular endothelial growth factor splice variants differentially regulate bone marrow-derived mesenchymal stem cells. J Cell Physiol 216: 458–468. 18288639 10.1002/jcp.21414

[36] Matsumoto R, Omura T, Yoshiyama M, Hayashi T, Inamoto S, et al (2005) Vascular endothelial growth factor-expressing mesenchymal stem cell transplantation for the treatment of acute myocardial infarction. Arterioscler Thromb Vasc Biol 25: 1168–1173. 15831811 10.1161/01.ATV.0000165696.25680.ce

[37] Al-Khaldi A, Eliopoulos N, Martineau D, Lejeune L, Lachapelle K, et al (2003) Postnatal bone marrow stromal cells elicit a potent VEGF-dependent neoangiogenic response in vivo. Gene Ther 10: 621–629. 12692590 10.1038/sj.gt.3301934

[38] Iwashita N, Uchida T, Choi JB, Azuma K, Ogihara T, et al (2007) Impaired insulin secretion in vivo but enhanced insulin secretion from isolated islets in pancreatic beta cell-specific vascular endothelial growth factor-A knock-out mice. Diabetologia 50: 380–389. 17180351 10.1007/s00125-006-0512-0

[39] Nakayama S, Uchida T, Choi JB, Fujitani Y, Ogihara T, et al (2009) Impact of whole body irradiation and vascular endothelial growth factor-A on increased beta cell mass after bone marrow transplantation in a mouse model of diabetes induced by streptozotocin. Diabetologia 52: 115–124. 18946656 10.1007/s00125-008-1172-z

[40] Sakata N, Chan NK, Chrisler J, Obenaus A, Hathout E (2010) Bone marrow cell cotransplantation with islets improves their vascularization and function. Transplantation 89: 686–693. 20101199 10.1097/TP.0b013e3181cb3e8d PMC2844476

[41] Duffy GP, Ahsan T, O'Brien T, Barry F, Nerem RM (2009) Bone marrow-derived mesenchymal stem cells promote angiogenic processes in a time- and dose-dependent manner in vitro. Tissue Eng Part A 15: 2459–2470. 19327020 10.1089/ten.TEA.2008.0341

[42] Tamama K, Sen CK, Wells A (2008) Differentiation of bone marrow mesenchymal stem cells into the smooth muscle lineage by blocking ERK/MAPK signaling pathway. Stem Cells Dev 17: 897–908. 18564029 10.1089/scd.2007.0155 PMC2973839

[43] Yue WM, Liu W, Bi YW, He XP, Sun WY, et al (2008) Mesenchymal stem cells differentiate into an endothelial phenotype, reduce neointimal formation, and enhance endothelial function in a rat vein grafting model. Stem Cells Dev 17: 785–793. 18522495 10.1089/scd.2007.0243

[44] Gong Z, Niklason LE (2008) Small-diameter human vessel wall engineered from bone marrow-derived mesenchymal stem cells (hMSCs). FASEB J 22: 1635–1648. 18199698 10.1096/fj.07-087924 PMC2605790

[45] Forte A, Finicelli M, Mattia M, Berrino L, Rossi F, et al (2008) Mesenchymal stem cells effectively reduce surgically induced stenosis in rat carotids. J Cell Physiol 217: 789–799. 18690654 10.1002/jcp.21559

[46] Quevedo HC, Hatzistergos KE, Oskouei BN, Feigenbaum GS, Rodriguez JE, et al (2009) Allogeneic mesenchymal stem cells restore cardiac function in chronic ischemic cardiomyopathy via trilineage differentiating capacity. Proc Natl Acad Sci U S A 106: 14022–14027. 19666564 10.1073/pnas.0903201106 PMC2729013

[47] Wang Q, Jin T (2009) The role of insulin signaling in the development of beta-cell dysfunction and diabetes. Islets 1: 95–101. 21099255 10.4161/isl.1.2.9263

[48] Matsuoka TA, Kaneto H, Miyatsuka T, Yamamoto T, Yamamoto K, et al (2010) Regulation of MafA expression in pancreatic beta-cells in db/db mice with diabetes. Diabetes 59: 1709–1720. 20424231 10.2337/db08-0693 PMC2889771

[49] Assmann A, Hinault C, Kulkarni RN (2009) Growth factor control of pancreatic islet regeneration and function. Pediatr Diabetes 10: 14–32. 18828795 10.1111/j.1399-5448.2008.00468.x PMC2630373

[50] Tuttle RL, Gill NS, Pugh W, Lee JP, Koeberlein B, et al (2001) Regulation of pancreatic beta-cell growth and survival by the serine/threonine protein kinase Akt1/PKBalpha. Nat Med 7: 1133–1137. 11590437 10.1038/nm1001-1133

[51] Gu Y, Lindner J, Kumar A, Yuan W, Magnuson MA (2011) Rictor/mTORC2 is essential for maintaining a balance between beta-cell proliferation and cell size. Diabetes 60: 827–837. 21266327 10.2337/db10-1194 PMC3046843

[52] Andjelkovic M, Alessi DR, Meier R, Fernandez A, Lamb NJ, et al (1997) Role of translocation in the activation and function of protein kinase B. J Biol Chem. 272: 31515–31524. 10.1074/jbc.272.50.31515 9395488

[53] Brazil DP, Hemmings BA (2001) Ten years of protein kinase B signalling: a hard Akt to follow. Trends Biochem Sci 26: 657–664. 11701324 10.1016/s0968-0004(01)01958-2

[54] Federici M, Hribal M, Perego L, Ranalli M, Caradonna Z, et al (2001) High glucose causes apoptosis in cultured human pancreatic islets of Langerhans: a potential role for regulation of specific Bcl family genes toward an apoptotic cell death program. Diabetes 50: 1290–1301. 11375329 10.2337/diabetes.50.6.1290

[55] Gratton JP, Morales-Ruiz M, Kureishi Y, Fulton D, Walsh K, et al (2001) Akt down-regulation of p38 signaling provides a novel mechanism of vascular endothelial growth factor-mediated cytoprotection in endothelial cells. J Biol Chem 276: 30359–30365. 11387313 10.1074/jbc.M009698200

